# Developing a Scorecard to Assess Global Progress in Scaling Up Diarrhea Control Tools: A Qualitative Study of Academic Leaders and Implementers

**DOI:** 10.1371/journal.pone.0067320

**Published:** 2013-07-09

**Authors:** Alexander Anthony Rosinski, Steven Narine, Gavin Yamey

**Affiliations:** 1 Global Health Sciences, University of California San Francisco, San Francisco, California, United States of America; 2 Evidence to Policy Initiative, Global Health Group, University of California San Francisco, San Francisco, California, United States of America; University of Western Australia, Australia

## Abstract

**Background:**

In 2010, diarrhea caused 0.75 million child deaths, accounting for nearly 12% of all under-five mortality worldwide. Many evidence-based interventions can reduce diarrhea mortality, including oral rehydration solution (ORS), zinc, and improved sanitation. Yet global coverage levels of such interventions remain low. A new scorecard of diarrhea control, showing how different countries are performing in their control efforts, could draw greater attention to the low coverage levels of proven interventions.

**Methods:**

We conducted in-depth qualitative interviews with 21 experts, purposively sampled for their relevant academic or implementation expertise, to explore their views on (a) the value of a scorecard of global diarrhea control and (b) which indicators should be included in such a scorecard. We then conducted a ranking exercise in which we compiled a list of all 49 indicators suggested by the experts, sent the list to the 21 experts, and asked them to choose 10 indicators that they would include and 10 that they would exclude from such a scorecard. Finally, we created a “prototype” scorecard based on the 9 highest-ranked indicators.

**Results:**

Key themes that emerged from coding the interview transcripts were: a scorecard could facilitate country comparisons; it could help to identify best practices, set priorities, and spur donor action; and it could help with goal-setting and accountability in diarrhea control. The nine highest ranking indicators, in descending order, were ORS coverage, rotavirus vaccine coverage, zinc coverage, diarrhea-specific mortality rate, diarrhea prevalence, proportion of population with access to improved sanitation, proportion with access to improved drinking water, exclusive breastfeeding coverage, and measles vaccine coverage.

**Conclusion:**

A new scorecard of global diarrhea control could help track progress, focus prevention and treatment efforts on the most effective interventions, establish transparency and accountability, and alert donors and ministries of health to inadequacies in diarrhea control efforts.

## Introduction

The World Health Organization (WHO) and the United Nations Children’s Fund (UNICEF) estimate that in 2010 diarrhea killed 751,000 children under five years, making it the second leading cause of childhood deaths worldwide after pneumonia [1]. Interventions to prevent diarrhea, particularly clean water, hand-washing with soap, improved sanitation, hygiene, breastfeeding, vitamin A supplementation, and rotavirus vaccination, can reduce mortality attributed to diarrheal disease [2]. Deaths from diarrhea can also be reduced by case management with oral rehydration solution (ORS) and zinc, and by treatment of dysentery with antibiotics [3]. A recent analysis using the Lives Saved Tool, a computer-based decision-making tool, found that scaling up these seven preventive interventions and three therapeutic interventions to near-universal coverage in 68 high child mortality countries would reduce diarrhea mortality by 92% at a cost of only $0.80 per capita [4].

Yet coverage levels of these preventive and therapeutic diarrhea control tools remain low [5]. For example, median coverage of improved sanitation in the 68 countries where over 95% of all child deaths occur worldwide is currently only about 40% [6]. Across these 68 high child mortality countries, the prevalence of hand-washing with soap ranges from only 3% to 42% [4]. And even though the WHO and UNICEF made a joint recommendation in 2004 that all children with diarrhea should receive ORS and zinc, less than 34% receive ORS and less than 1% receive zinc in developing countries [5], [7].

A key reason for such low coverage rates is the lack of global attention, advocacy, and funding for diarrhea control, particularly when compared with the control of other childhood diseases, such as malaria. Attention to diarrhea control among the global health community has dramatically declined since the 1980s, a decade in which there was a major global push to scale up ORS [8]. Bump and colleagues analyzed the level of priority that diarrhea has been given on the global health agenda from the 1970s to today. Using four measures of priority—trends in treatment coverage, changes in perceived priority, changes in financial support and institutional involvement, and bibliographical trends—they found that today the “global level priority of diarrheal disease is about one sixth to one third as high as in 1985” [9]. A qualitative study involving over 50 key diarrhea stakeholders worldwide concluded that there is “an overwhelming consensus that attention and momentum around diarrheal disease have stalled, and that increased advocacy is critical for re-prioritizing the issue” [10].

Diarrhea control continues to be a much lower funding priority among donors and ministries of health compared to combating malaria, HIV, tuberculosis, and other illnesses that have a lower burden of mortality than diarrhea. For example, the two largest malaria donors are the Global Fund to Fight AIDS, Tuberculosis and Malaria (GFATM) and the United States President’s Malaria Initiative (PMI); in 2010, GFATM spent $630 million on malaria treatment and in 2011 PMI spent $104 million on malaria treatment [11]. In contrast, the largest procurer of ORS and zinc is UNICEF; in 2010, its Supply Division spent just $2.3 million on the purchase of zinc tablets and $4.9 million on the purchase of ORS sachets (Mark Young, UNICEF, personal communication). Excluding such commodity costs, UNICEF’s entire childhood diarrhea control program allocation was only about $24 million in 2011, comprising $12 million for regular diarrhea programming and $12 million for programming in emergency and humanitarian settings including cholera control (Mark Young, UNICEF, personal communication).

Diarrhea also receives less media attention than other childhood diseases. Hudacek and colleagues compared newspaper coverage of the three GFATM diseases with coverage of three high-burden childhood diseases (pneumonia, diarrhea, and measles) between 1981 and 2008 [12]. The GFATM diseases attracted almost 5 times as much newspaper coverage as the other three diseases (1,344,150 versus 291,865 articles) [12]. And a much greater proportion of funding for research and development is allocated to HIV/AIDS, malaria, and tuberculosis than to diarrheal diseases [13].

One tool that could potentially help to increase global attention to diarrhea control is a “scorecard” or index that compares how different countries are performing against each other in scaling up diarrhea control tools. Scorecards are increasingly being used in global health and development. Such scorecards can be helpful not only for cross-country comparisons, but also for tracking how a specific country is performing over time in reducing the disease burden through scale up of control tools and adoption of supportive national policies. Davis and Kingsbury, in a report for the Rockefeller Foundation, have argued that such scorecards “stimulate and shape action by alerting people to the existence of a problem, helping them to understand its magnitude, and pointing toward appropriate interventions” [14].

For example, in September 2011, the African Leaders Malaria Alliance (ALMA), an alliance of African heads of state and government working to control malaria, launched the Scorecard for Accountability and Action, which is updated quarterly [15]. The scorecard tracks how 40 different malaria-endemic countries are performing against a set of indicators of malaria control, such as national coverage with insecticide-treated bed nets and removal of tariffs on malaria control tools [16]. The scorecard is reviewed biannually at ALMA meetings by ALMA heads of state and government [15].

Other examples of such scorecards or indexes that have been recently launched, with their year of launch shown in parentheses, include: The London Declaration for Neglected Tropical Diseases Scorecard (2013), the Infant and Toddler Feeding Scorecard (2012), the Commitment to Vaccination Index (2012), the Aid Transparency Index (2010), the Access to Medicine Index (2008), and the Ibrahim Index of African Governance (2007) [17]–[22]. These scorecards often use simple and compelling graphics that allow readers to quickly visualize how countries stack up against each other in achieving a particular health or development goal. Some scorecards assign different “traffic light” colors to each country to indicate whether the country is not on target (red), on target (yellow), or has achieved the target (green) [15], [17].

In this new qualitative study, involving key informant interviews with internationally recognized child health and diarrhea experts, we explored the opportunities for, and barriers to, using a scorecard as a policy tool for increasing the use of key preventive and therapeutic diarrhea control tools. We also investigated experts’ views on which indicators should be included in such a scorecard and used a ranking exercise to prioritize the suggested indicators in order to create a “prototype” scorecard.

## Methods

### (a) Qualitative Study

We conducted individual in-depth interviews with 21 experts to explore their views on how a diarrhea control scorecard might influence policy-making by ministries of health and international health agencies and on which indicators should be included in such a scorecard. The key informants (KIs) were purposively sampled based upon their established expertise in diarrhea and child health [23]. These experts were chosen based on a combination of (a) their research publication record (i.e., they had published extensive research related to childhood diarrhea), and (b) their academic leadership or implementation expertise in the field of global diarrhea control. We also used a snowballing technique, in which we asked each of the interviewees to suggest additional experts [24].

Three interviews were conducted in person and 18 by telephone, using a semi-structured interview guide (Text S1). At the time that the interviews were conducted, 14 KIs were based in high-income countries (HICs), one was based in a low-income country (LIC), and 6 divided their time between HICs and LICs. Eleven KIs focused primarily on diarrhea treatment and prevention in their research or public health practice, while five KIs focused primarily on water and sanitation. Our sample of 21 KIs included 13 academic researchers and 6 “technical advisors” who are responsible for leading and implementing diarrhea control programs in low- and middle-income countries (LMICs). Table S1 gives the KIs’ demographic information, including their current positions and brief descriptions of their publication records. In order to protect KIs’ anonymity, identifying information has been excluded from Table S1 and from the Results section of this paper.

All interviews were tape recorded and transcribed. Two researchers (AR, SN) independently coded the interview transcripts, using a grounded theory approach, and they resolved any differences through discussion and consensus [25]. Theoretical saturation was reached by the nineteenth interview [26].

### (b) Ranking of Indicators

One of the questions in the qualitative study was: “Which indicators or measures do you believe should be included in a scorecard of global diarrhea control?” (Text S1). A total of 49 different indicators were suggested by the KIs. In the second stage of our study, we sent this list of all 49 suggested indicators to the 21 KIs, by e-mail, and asked them to select 10 indicators that they think should be included in such a scorecard and 10 indicators that they would exclude. In order to improve the response rate, KIs who did not respond to our first e-mail request were contacted up to two more times.

The list of indicators and the specific wording of our request to choose or exclude indicators is shown in Text S2. For convenience, the 49 indicators were grouped into 7 categories: water and sanitation indicators; indicators related to coverage of ORS and zinc; indicators of vaccine coverage; maternal and child health indicators; indicators related to ministry of health policies; indicators related to the social determinants of health; and indicators of the diarrheal disease burden. We pre-tested text S2 for clarity on three professors working in global health at University of California, San Francisco; all three understood what was being asked and were able to choose 10 indicators and exclude 10 indicators in about 3–5 minutes.

After receiving the responses from the KIs, we ranked the indicators using a simple point system. One point was awarded to an indicator every time that it was chosen for inclusion in the scorecard by a KI; one point was subtracted from an indicator every time that a KI excluded it from the scorecard. At the end of this process, we calculated a final score for each indicator and ranked the 49 indicators from highest to lowest score.

### (c) Creating a Scorecard Prototype

Finally, we included the 9 highest scoring indicators in a “prototype” scorecard. The prototype shows how the 15 countries with the highest burden of childhood diarrhea mortality are performing in their diarrhea control efforts as judged by these 9 indicators.

The decision to use the 9 highest-ranking indicators was based on the results of the qualitative study: KIs indicated that the scorecard should be kept simple with a manageable number of indicators. We organized the indicators on the prototype scorecard into four categories—treatment, prevention, protection, and impact. Again, such organization was guided by the results of the qualitative study.

The fifteen countries with the highest number of child deaths due to diarrhea in 2007 were selected for inclusion in the prototype scorecard [3]. Country-level data on how these 15 countries are performing on the 9 indicators included in our prototype scorecard were obtained from the sources shown in Table 1. Diarrhea prevalence figures were taken from the most recent demographic and health survey (DHS) report available, shown in Table 2. These surveys, which can be downloaded at http://www.measuredhs.com/, are nationally-representative household surveys, supported by the United States Agency for International Development, carried out by ICF Macro/MEASURE DHS on behalf of national ministries of health.

**Table 1:** Indicator definitions and sources of country-level data in the prototype scorecard.

Indicator	Indicator Definition	Indicator Year	Source
ORS coverage	% of children under age 5 years with diarrhea receiving ORS	Various	UNICEF 2012 [5]
Zinc coverage	% of children under age 5 years with diarrhea receivingzinc treatment	Various	UNICEF 2012 [5]
Rotavirus vaccine coverage	% of infants who received the complete rotavirusvaccine series	2012	PATH [27]
Exclusive breastfeeding	% of infants <6 months who were exclusively breastfed	Various	UNICEF 2012 [5]
Measles vaccine coverage	% of one-year-old children immunized against measles	2010	UNICEF 2012 [5]
Improved water	% of population with access to an improved water source	2010	WHO/UNICEF [28]
Improved sanitation	% of population with access to an improved sanitation facility	2010	WHO/UNICEF [28]
Diarrhea prevalence	% of children under age 5 who had diarrhea in thetwo weeks preceding the survey	Various	Most Recent DHS [29]; see Table 2
Diarrhea-specific mortality rate	Number of deaths in children under 5 per 1,000 live births	2010	WHO [30]

**Table 2:** Most recent DHS used for diarrhea prevalence indicator.

Country^a^	Most recent DHS [29]
Afghanistan	Standard DHS unavailable
Angola	Standard DHS unavailable
Bangladesh	2007
Burkina Faso	2010
China	Standard DHS unavailable
Democratic Republic of the Congo	2007
Ethiopia	2011
India	2005–2006
Kenya	2008–2009
Mali	2006
Niger	2006
Nigeria	2008
Pakistan	2006–2007
Uganda	2011
United Republic of Tanzania	2010

a Countries listed in alphabetical order.

### Ethical Considerations

This study was approved by the University of California, San Francisco Committee on Human Research. The committee approved the use of oral consent, as this study was classified as “minimal risk research.”

We took steps to protect the privacy and confidentiality of the participants: no identifying information is included in this study report and the KIs are referred to by an anonymized number only (ranging from KI 1 to KI 21).

## Results

### (a) Qualitative Study

We invited a total of 44 experts to participate in this study, of whom 21 agreed.

Six major themes related to a scorecard of global diarrhea control emerged from coding the transcripts of the interviews with these 21 experts.

#### Theme 1: A scorecard could facilitate country comparisons and stimulate competition

Three KIs (KIs 9, 11, 14) argued that there is currently no tool for readily cross-comparing different countries’ performance in controlling childhood diarrhea; instead, indicators of performance are scattered in “country profiles” featured in many different reports, making cross-comparison difficult. A new scorecard would allow cross-comparison of countries’ performance in controlling diarrhea in children (KIs 7, 8, 9, 11, 14), and it would be beneficial if it allows policymakers and government leaders to see where they stand compared to other countries with regards to diarrhea control (KIs 1, 8, 9, 11, 14). If such a scorecard were to be used, argued five KIs (1, 9, 13, 14, 18), ministries of health in LICs would care about where their own countries stand in relation to other countries, which could set up an element of healthy competition.

*“The scorecard lets leaders know relative to other countries in their region how they are doing, and it has this kind of rallying effect.”* (KI 1)

*“I think it can be useful in-country to have simple scorecards that help the program managers, leadership and ministry of health to prioritize and understand how their country compares to other countries. You know, that can be very motivating. And it can be a useful tool in-country to say, you know, set priorities and create urgency.”* (KI 9)

Fourteen of the 21 KIs raised the question of how countries would respond to poor scores on the scorecard. As with any ranking or assessment of performance, the scorecard would identify winners and losers (KIs 1, 2, 6, 11, 14). Twelve KIs (KIs 1, 4, 5, 6, 9, 10, 11, 13, 14, 16, 17, and 20) felt that low scores would motivate ministries of health and other stakeholders to take action, although two KIs expressed concern about the sensitivity around naming poor performers (KI 2, 8).

*“I think naming and shaming has been incredibly successful in global health. I think it’s widely accepted. Child immunization numbers really spurred countries to get sort of competitive with each other and get up the ranking. The corruption indexes, you know, when the corruption indexes come out every year or two, countries take heed. And I think health is particular because ministries of health, you know, are very often run by well-trained and well-intentioned people who know how important vaccination is or water supply or bed nets or HIV treatment or whatever it is. And so these indexes ring true to them. And I think generally, in my experience working with ministries of health, they don’t dismiss them [the numbers]; they take them seriously and try to do something about them.”* (KI 14)

*“As long as scorecards are used in a constructive way and they’re used internally, so you know, they’re an internally valid measure of how a particular country or a particular program is doing, I think I’m okay with that. When you start to move beyond that to say, well, this country is better than that country or is doing a better job and moving forward quicker, or whatever, then you’re going to start to get some sensitivities there.”* (KI 2)

*“The scorecard should be a tool to help countries do things better, not just a tool to make them feel like they’re crap.”* (KI 8)

#### Theme 2: A scorecard could help to identify best practices, set priorities, and spur donor action

The second major theme emerging from the qualitative study is that a scorecard could draw attention to countries that are successfully controlling diarrhea in children and could thus facilitate the sharing of best practices (KIs 1, 9, 11, 19).

*“Let’s say you see a country in Africa at 60% ORS coverage. And you say, ‘wow, what are they doing? What has been their strategy and approach for success?’ The scorecard would identify those, say, positive deviants so that then you can really do more detailed case studies and look for the lessons learned.”* (KI 11)

The scorecard could also call attention to low coverage levels of key evidence-based diarrhea control tools, such as zinc and rotavirus vaccination (KIs 1, 2, 4, 5, 6, 9, 10, 11, 13, 14, 16, 18). This in turn may prompt donors and ministries of health to align their resources with particular areas of need and to prioritize scale-up of specific diarrhea control interventions (KIs 1, 6, 8, 9, 11, 13, 14). For example, donors and non-governmental organizations (NGOs) may possibly see low coverage of zinc on the scorecard as an investment opportunity (KIs 1, 9, 13, 14).

*“If the scorecard gets the person that runs the child health program in Malawi to be like, ‘Oh jeez, we’re really far behind on diarrhea, we need to do something about this,’ that’s super valuable. It can be a useful tool in-country to say, set priorities and create urgency.”* (KI 9)

*“At a national level, [scorecards] are also useful because they might provide some either political support or technical justification, depending on how it is made for a policy decision, and that’s really helpful.”* (KI 6)

#### Theme 3: Use of a scorecard could help to monitor progress over time but better monitoring and evaluation data will be needed

A scorecard could be used to track progress on diarrhea control over time (KIs 1, 2, 4, 5, 11, 13, 20).

*“This [tracking progress on diarrhea control] is something that the WHO is obviously concerned about. You know, how do you monitor progress? How do you in fact document whether or not in terms of a Millennium Development Goal or a diarrhea disease control goal, whether you’re headed in the right direction or not. I think that’s important.”* (KI 2)

*“The purpose of a scorecard is to sort of—is to describe how much is left undone, and where we are now.”* (KI 1)

Three KIs (KIs 6, 9, 14) explained that the global health community currently lacks a tool for monitoring and evaluating progress on diarrhea.

*“Right now there’s no mechanism for measuring in a consolidated way where countries are on diarrhea control.”* (KI 9)

However, for a scorecard to be useful in tracking progress over time, better monitoring and evaluation data will be needed related to the burden of diarrhea and to coverage with diarrhea control tools (KIs 2, 6, 9, 10, 13, 16, 18).

*“I’m not surprised about [the lack of data on] diarrhea at all. I mean, you have to look at donor priorities. Where are current donor priorities? It’s not on diarrhea, so I’m not surprised that we are not improving measurement of diarrhea or associated treatment or prevention of diarrhea.”* (KI 18)

#### Theme 4: A scorecard could help with goal setting and accountability

Eight KIs (KIs 1, 2, 6, 9, 10, 11, 13, and 14) said that a scorecard could facilitate goal setting among ministries of health, NGOs, and other key stakeholders. These actors could agree on targets for each of the indicators on the scorecard (KIs 1, 10).

*“I think that scorecards help—you know, it helps to have an objective and it helps to have a clear path forward, you know. So we all kind of know more or less the direction we’re moving in and how we’re proceeding in achieving that.”* (KI 10)

KIs 1, 11, and 13 suggested that a “traffic light” color coding system could be used to indicate whether a country is “not on track” (red), “on track” (green), or “almost on track” (yellow) to achieve diarrhea control goals.

*“The [color coding system] actually takes a lot of thinking because you want to be really clear on what’s the gold standard. So what is your green? What is the ultimate goal? What would you consider success? And then sort of work backwards in a way and say, you know, what is the situation now in most countries and what’s the gold standard and then what’s in between. What’s that first hump that you have to get over in order to get the ball rolling, you know, that’s often where the threshold is between red and yellow, for instance.”* (KI 1)

However, KIs 1 and 13 mentioned that selecting thresholds for what is considered red, yellow, and green could be difficult.

*“Even in Bangladesh, Gates and others spent $7 million in Bangladesh over a five-year period to introduce zinc and get it out there, and they plateaued at 20 percent. So do you rank that as, you know, that’s a lot of money and a lot of investment. Do you rank that as, anything over 20 percent is green or anything over, you know, 15 to 30 percent is yellow? I don’t know.”* (KI 13)

A scorecard could also be used to establish accountability around commitments and pledges to combat childhood diarrhea (KIs 1, 9, 10, 13, 14). Three KIs (KIs 1, 9, 13) said that such accountability is especially relevant in light of the recent commitments that donors made at the 2012 Child Survival Call to Action summit (at which donors committed to reducing child mortality to below 20 child deaths per 1000 live births by 2035) (KIs 1, 9, 13).

*“[The scorecard] is intended to keep the accountability alive, you know, so that it’s not just, you know, a media interview or something like that where a ministry of health stands up and says ‘we’re committed to doing this,’ but instead it’s actually a tool that gets reviewed—it’s a reporting tool.”* (KI 1)

The scorecard could also establish transparency in countries’ efforts to control diarrhea in children (KI 1, 14).

*“If we had a diarrhea scorecard, we would know who was doing well and who was not doing well in anti-diarrhea work. And at the moment, I don’t think we know that.”* (KI 14)

#### Theme 5. Launching a scorecard would capitalize on global momentum on scaling up child health commodities

Six KIs (KIs 1, 9, 10, 13, 14, and 21) expressed that this study was timely given the newly established United Nations Commission on Life-Saving Commodities for Women and Children (established in March, 2012) and the recent Child Survival Call to Action summit (held in June, 2012).

*“There’s this growing global momentum around a couple key child health commodities that aren’t getting to the kids who need them that are cheap and available, like ORS, like zinc, like amoxicillin for pneumonia. Right now there’s no mechanism for measuring in a consolidated way where countries are on diarrhea control. So a scorecard could be hugely useful and timely in the global community right now if it’s focused on some of these child health medicines that are being focused on by the new UN Commission and in a number of countries.”* (KI 9)

#### Theme 6. A scorecard should be kept simple and targeted to its audience

Four KIs (KIs 1, 6, 13, and 19) suggested that there should be a manageable number of indicators on the scorecard, since each indicator is basically a recommendation to either scale up an intervention or to change policy. KIs 1 and 13 believed that if a scorecard included too many indicators, then these indicators would lose their importance.

*“I don’t know if there is a magic number, necessarily. I would say the fewer, the better. You know, something more in the 10–12 range is manageable. Because otherwise, you know, if they’re too overlapping or if they’re too detailed you lose the meaning of it. It should really matter whether one of those cells is in the red.”* (KI 13).

In addition, KIs 1, 5, 8, and 21 suggested that indicators on the scorecard should be changeable within a short-term time frame.

*“Look at it from the lens of what you would expect to change in one year’s time, you know, because you don’t want to put indicators that will take ten years to move from yellow to green. You know, you want to set the indicators such that they are actionable and really indicate progress.”* (KI 1)

Five KIs (KIs 6, 12, 13, 16, 21) mentioned that ministries of health and NGOs should be capable of influencing and moving the indicators on the scorecard.

*“I think it would give the most value if the indicators that are on the scorecard are also really manageable—directly manageable for programs to improve on. For example, maternal education, it’s really hard to improve on that. We really like indicators that are within the scope of the ministry of health—within their reach to significantly improve on.”* (KI 13)

*“You’d like the scorecard to be adjusted for the possible actions that the audience could take. If you said the scorecard is for, let’s say, district officials, then I would say the scorecard should focus on things they could change, which might mean ORS, zinc, rotavirus vaccine coverage, vitamin A, maybe some behaviors like hand-washing, latrine usage. But at that level they’re probably not empowered or in a position to get infrastructure change; whereas, at the global level, infrastructure ought to be on the list.”* (KI 6)

However, four KIs (2, 5, 11, and 20) felt that maternal education level and other social determinants of health should be included on the scorecard, since these indicators are strong predictors of whether or not a child will die of diarrhea.

*“Clearly, social determinants of disease can’t be ignored. They’re rather indirect but, you know, the level of proportion of women who receive secondary education is probably as good as an indicator of what’s going to happen in the future in terms of health outcomes, including diarrhea, as anything you’re going to get.”* (KI 2)

### (b) Ranking of Indicators

#### Indicators proposed by key informants

The 21 KIs proposed a total of 49 indicators that they believed would be suitable for inclusion in a scorecard of global diarrhea control (Table 3).

**Table 3:** Indicators proposed by the 21 KIs for inclusion in a scorecard of diarrhea control.

Category	Indicator
**Water and sanitation indicators**	% of population with access to improved sanitation facilities
	% of population practicing open defecation
	% of population with access to improved drinking water
	An indicator of hand-washing rates
	An indicator on latrine usage
	Whether or not a country has implemented a mass media campaign to promote hand-washing
	% of schools with access to latrines for boys and girls separately
	Proportion of urban households connected to sewage
	An indicator of urban versus rural latrine usage
**Indicators related to coverage of oral rehydration solution** **(ORS) and zinc**	Zinc coverage in children under five with diarrhea
	ORS coverage in children under five with diarrhea
	no. of districts where zinc and low-osmolarity ORS are available
	% of retailers carrying ORS and zinc
	no. of local pharmaceutical manufacturers producing zinc and ORS in the country
	% of mothers between the ages of 15 and 45 who know that zinc and ORS are appropriate treatments for diarrhea
	An indicator of zinc availability in the public sector
	An indicator of zinc availability in the private sector
	Proportion of diarrhea cases seen in health facilities that were treated with both zinc and ORS
	Whether or not there are ORS/zinc co-packaged products
	% of providers correctly administering or prescribing diarrhea treatment
**Indicators of vaccine coverage**	Year of introduction of pneumococcal vaccine
	Pneumococcal vaccine coverage
	Measles vaccine coverage
	Meningococcal vaccine coverage
	Haemophilus vaccine coverage
	Influenza vaccine coverage
	Pertussis vaccine coverage
	Rotavirus vaccine coverage
**Maternal and child health indicators**	% of under-fives with pneumonia taken to an appropriate healthcare provider
	% of under-fives with diarrhea taken to an appropriate healthcare provider
	Coverage with exclusive breastfeeding
	Bed net coverage among children
	Coverage with interventions for prevention of mother to child transmission of HIV (PMTCT)
	Coverage with family planning
	Vitamin A coverage in children
	Height for age Z score at second birthday
**Indicators related to ministry of health (MoH) policies**	Whether or not a country has over-the-counter (OTC) status for zinc
	Whether or not the MoH plans to introduce rotavirus vaccination
	Total funding for diarrhea from government and external donors
	An indicator of how well the Water, Sanitation, and Hygiene (WASH) and treatment sectors are integrated
	Number of ORS packets funded as a percentage of ORS packets needed to have full coverage
	% of MoH workers trained in the new diarrhea management protocols under Integrated Management of Childhood Illness (IMCI)
	Number of months with stock outs of ORS and zinc in public health facilities
	Presence of community case management protocols for diarrhea that include ORS and zinc
	Whether or not the public sector purchases ORS and zinc (versus relying on donations)
**Indicators related to the social determinants of health**	% of women who receive secondary education
	% of seats held by women in the national government
**Indicators of the diarrheal disease burden**	Diarrhea prevalence
	Diarrhea-specific mortality rate

#### Results of ranking exercise

Sixteen out of the 21 KIs responded to our request to choose 10 indicators that they would include and 10 that they would exclude from a diarrhea control scorecard (a 76% response rate). The results of the ranking exercise are shown in Table 4. Only 8 of the 16 KIs that completed the questionnaire selected both 10 indicators for inclusion on a global diarrhea control scorecard and 10 indicators for exclusion. While 13 of 16 KIs marked exactly 10 indicators for inclusion, only 8 of 16 KIs marked exactly 10 indicators to exclude from the scorecard.

**Table 4:** Ranking of indicators to include on a scorecard of global diarrhea control.

Rank	Indicator^a^	Score
1	ORS coverage in children under five with diarrhea	15
2	Rotavirus vaccine coverage	11
3	Zinc coverage in children under five with diarrhea	10
T4	Diarrhea-specific mortality rate	9
T4	Diarrhea prevalence	9
T4	% of population with access to improved sanitation facilities	9
T5	% of population with access to improved drinking-water	7
T5	Coverage with exclusive breastfeeding	7
6	Measles vaccine coverage	6
T7	An indicator of hand-washing rates	4
T7	% of population practicing open defecation	4
T7	Whether or not the MoH plans to introduce rotavirus vaccination	4
T7	Proportion of diarrhea cases seen in health facilities that were treated with both zinc and ORS	4
T8	Whether or not a country has over-the-counter (OTC) status for zinc	3
T8	Vitamin A coverage in children	3
T8	% of women who receive secondary education	3
T9	Proportion of urban households connected to sewage	2
T9	% of mothers between the ages of 15 and 45 who know that zinc and ORS are appropriate treatments for diarrhea	2
T9	Presence of community case management protocols for diarrhea that include ORS and zinc	2
T9	An indicator on latrine usage	2
T10	Height for age Z score at second birthday	1
T10	% of MoH workers trained in the new diarrhea management protocols under Integrated Management of Childhood Illness (IMCI)	1
T10	Total funding for diarrhea from government and external donors	1
T10	% of providers correctly administering or prescribing diarrhea treatment	1

a Only indicators that received 1 point or more were included in Table 4

### (c) Scorecard Prototype

The scorecard prototype (Table 5) includes the nine highest ranking indicators: rotavirus vaccine coverage, zinc coverage, diarrhea-specific mortality rate, diarrhea prevalence, proportion of population with access to improved sanitation, proportion with access to improved drinking water, exclusive breastfeeding coverage, and measles vaccine coverage. Table 5 shows coverage levels for the seven “intervention” indicators (i.e., treatment, prevention, and protection indicators), diarrhea prevalence rates, and diarrhea-specific mortality rates in the 15 countries with the highest childhood diarrhea mortality burden.

**Table 5:** Global diarrhea control scorecard prototype.

	Treatment		Prevention			Protection		Impact	
Country^a^	ORS coverage(Various)	Zinccoverage(Various)	Rotavirus vaccine coverage(2012)	Exclusive breastfeeding(Various)	Measles vaccine coverage(2010)	Improved water(2010)	Improved sanitation(2010)	Diarrhea prevalence(Various)	Diarrhea-specific mortality rate(2010)
Afghanistan	30	No data	<1	No data	62	50	37	No data	23.84
Angola	40	No data	<1	11	93	51	58	No data	24.15
Bangladesh	77	23	<1	43	94	81	56	10	2.88
Burkina Faso	17	No data	<1	16	94	79	17	14.7	21.12
China	No Data	No data	<1	28	99	91	64	No data	0.54
DRC^b^	26	2	<1	37	68	45	24	16.4	22.1
Ethiopia	26	No data	<1	49	81	44	21	13.4	14.84
India	26	<1	<1	46	74	92	34	9	8.19
Kenya	39	<1	<1	32	86	59	32	16.6	7.65
Mali	14	No data	<1	38	63	64	22	13.3	24.92
Niger	18	No data	<1	27	71	49	9	20.8	20.02
Nigeria	26	1	<1	13	71	48	9	10.1	15.73
Pakistan	41	No data	<1	37	86	92	48	21.8	9.57
Tanzania^c^	44	5	No data	50	92	53	10	15	6.84
UgandaData Source	40UNICEF 2012 [5]	1UNICEF 2012 [5]	<1PATH [27]	60UNICEF 2012 [5]	55UNICEF 2012 [5]	72WHO/UNICEF [28]	34WHO/UNICEF [28]	23.4Most recent DHS [29]	9.9WHO [30]

a Countries listed in alphabetical order.

b DRC: Democratic Republic of the Congo.

c Tanzania: United Republic of Tanzania.

## Discussion

### Principal Findings

Diarrhea control and child health experts interviewed in this qualitative study believed that it would be feasible to develop a scorecard as a policy tool for guiding diarrhea control strategies. These experts felt that such a tool, provided it was simple and non-burdensome, could be a valuable mechanism for stimulating healthy competition between countries to reduce their diarrhea burden, for facilitating the sharing of best practices, for tracking progress over time, and for establishing a global diarrhea control accountability mechanism. The qualitative study also suggested that launching a diarrhea control scorecard at this time would capitalize on recent global momentum towards taking action on childhood diarrhea. By using a ranking exercise, we were able to create a simple prototype scorecard showing how the 15 countries with the highest childhood diarrhea burden are performing on the nine highest-ranked indicators.

### Strengths and Weaknesses of the Study

To the best of our knowledge, this is the first study to examine experts’ views on the value and development of a diarrhea control scorecard, and also the first to develop a “prototype” scorecard. One previous study, by Kosek and colleagues, used a Delphi process in which 10 diarrhea experts were asked to rank research investment priorities for reducing the global diarrhea burden [31], but our study focus was on current indicators of progress rather than on how future research efforts should be directed.

In addition to its novelty, another strength of our study is that it included a very diverse range of key informants, including experts from academia, donor organizations, think tanks, and health consulting companies, many of whom have deep expertise and “on the ground” experience in diarrhea control activities. The findings of the study are therefore likely to represent “real world” views, rather than purely theoretical or “ivory tower” views, on the value and construction of a diarrhea control scorecard.

We believe that creating a “prototype” scorecard was valuable because it will help to stimulate discussion and debate in the global health community. However, the scorecard developed in this study remains at an early stage of development, and will need further refinement and validation before it could be used in practice. But we have nevertheless shown that creating a scorecard is feasible and that there is widespread support among the diarrhea and child health experts that we interviewed for the value of such a scorecard.

Scorecards commonly assign different “traffic light” colors to each indicator showing how a country is progressing towards a target (e.g., a target of 100% coverage with insecticide-treated bed nets as a malaria prevention tool) [15], [17], [18]. These demarcations or thresholds (red for “off target,” yellow for “on target” and green for “target has been achieved”) should ideally be based on scientific data and consensus. We chose not to designate such thresholds on our prototype scorecard since our study did not explore the data or scientific consensus views on where such thresholds should be set. The main purpose of our prototype scorecard is to provide a visual example of a scorecard of global diarrhea control, based on the results of our qualitative study and ranking exercise. Future research could be directed at defining demarcations or thresholds to visually represent how countries are progressing towards specified targets.

One important limitation of this study was the under-representation of KIs from LICs, including ministers of health. Although six experts from ministries of health in LICs were contacted, none chose to participate in this study. While our KIs believed that the scorecard would be useful to ministries of health, we were not afforded the opportunity to directly hear from the ministers themselves. It is possible that ministers of health would have provided a different perspective on the scorecard. For example, ministers may have expressed concerns about the way in which a scorecard could “name and shame” certain countries. They also may have suggested different indicators to be included in the study.

Twenty of the 21 KIs interviewed in our study were based in HICs at the time of the interviews. However, 19 of our 21 interviewed experts have had recent and extensive experience working on childhood diarrhea control in LMICs. It is still possible that interviews with experts who are currently based in LMICs would have revealed a different set of opportunities and barriers to using the scorecard as a policy tool. As one of us (GY) has previously argued, qualitative studies in global health that interview experts who are based mostly in rich countries, particularly experts based in donor agencies, can give a biased view of solutions to health challenges [32]. As Yamey says: ‘in malaria control, for example, they [donors and international agencies] tend to favor a “visible quick-fix solution” over more complex approaches” [32].

Our sample of KIs may also have introduced bias with regards to areas of expertise. Ten of the KIs were experts in ORS and zinc, whereas only five of the KIs were experts in water and sanitation. Such bias could explain why the highest ranked indicators were more heavily focused on diarrhea treatment than on water and sanitation.

### Relationship to Previous Research

Although scorecards and indexes have recently become widely used in global health and development, there has been little research on their development, use, performance, validity, or impact. In their report on the use of scorecards in global development, Davis and Kingsbury note that “surprisingly little work has been done” on understanding the effects of scorecards, especially in global governance and transnational contexts relevant to development [14]. The authors summarize several case studies of scorecards, including the global immunization coverage indicators produced by the WHO and UNICEF, but empirical research on such scorecards remains rudimentary.

The Balanced Scorecard, a data-driven assessment and management tool used extensively within businesses since the 1990s, has been studied and recently applied in hospitals and by ministries of health [33]. For example, in 2004, the Ministry of Public Health in Afghanistan developed a Balanced Scorecard to “regularly monitor the progress of its strategy to deliver a basic package of health services,” which included “29 core indicators and benchmarks representing six different domains of health services, together with two composite measures of performance” [34]. The six health services domains in the scorecard are patients and community; staff; capacity for service provision; service provision; financial systems; and overall vision [34]. In 2007, Peters and colleagues described how the scorecard was created, how it is used, and the first results of its use; they conclude that the scorecard has been a useful tool for the ministry, NGOs, and other stakeholders, and has become “one of the cornerstones of the government’s monitoring and evaluation system” [34]. Balanced Scorecards applied to public health have since been studied extensively; however, these scorecards are generally used internally on an organizational or national level and are not intended to facilitate country or cross-organizational comparisons.

Scorecards and indexes have sometimes been published with accompanying reports that explain their underlying methodology. For example, annual reports accompany the Access to Medicine Index and the Aid Transparency Index that explain how and why indicators were selected and their methods of data collection [20], [21]. Such explanatory materials that outline the rationale and criteria for selecting indicators could aid in the development of other scorecards or indexes. However, while many scorecards use a “traffic light” threshold system, there is rarely an explanation for how these threshold levels were chosen. For example, neither the ALMA Scorecard for Accountability and Action nor the London Declaration for Neglected Tropical Diseases Scorecard give explanations for how certain thresholds were chosen.

There has, however, been substantial research on ranking disease control priorities for improving global health and development and on ranking health research priorities for specific diseases. These studies have often involved a Delphi process in which experts generate and then rank a list of priorities. In the 2012 Copenhagen Consensus, for example, a panel of economists ranked the interventions that they believed could have the greatest impact on global development (the highest ranking intervention was “bundled micronutrient interventions to fight hunger and improve education”) [35].

Such ranking exercises do have similar goals to scorecards: both help to advocate for specific interventions, and indeed they can have synergistic effects in raising awareness. For example, in Kosek and colleagues’ study, in which experts ranked diarrhea research priorities, the three highest ranked priorities were: (1) research to improve the deliverability and cost of zinc treatment, (2) cost-effectiveness studies of the rotavirus vaccine, and (3) health policy and systems research to increase access to ORS packets at all times in all sites for all children who may need it [31]. In our study, ORS coverage, rotavirus vaccine coverage, and zinc coverage were also the three highest ranked indicators. Thus the research ranking and our scorecard together can help to shine a spotlight on the urgent need to scale up these three key interventions.

Previous trials and systematic reviews have helped to define interventions of high efficacy in reducing diarrhea mortality in children. For example, a 2009 UNICEF/WHO report on diarrhea laid out a package of key diarrhea interventions that included clean water, sanitation, rotavirus and measles vaccines, breastfeeding, vitamin A supplementation, and ORS and zinc [3]. Our nine top-ranked indicators show major overlap with this package, although vitamin A supplementation did not rank in the top nine (Table 4 shows the ranking of childhood vitamin A coverage). Similarly, while a recent Lives Saved Tool analysis that listed key diarrhea interventions included antibiotics for dysentery, hand-washing with soap, and vitamin A [4], these interventions were not included in our final scorecard. There are a number of potential explanations for these discrepancies. One possible reason is that KIs chose interventions that they considered to be “low hanging fruit” (those that are easier to scale up quickly in the real world) [32]. For example, despite good evidence on efficacy [44]–[45], the Global Scaling Up Handwashing Project notes that “large-scale promotion of handwashing behavior change is a challenge” [46]. Another explanation is that the ranking reflected the composition of KI expertise.

### Policy Implications and Next Steps

We believe that our study has three key policy implications.

First, diarrhea continues to be underfunded and under-prioritized relative to its burden, and a scorecard of global diarrhea control could help to raise the priority of this disease and of action towards its control. A scorecard could draw greater attention among the global health community to diarrhea deaths and alert ministries of health and donors to inadequacies in diarrhea control efforts. Poor scores on the scorecard may “name and shame” ministries of health and government leaders into taking action. A scorecard could prompt stakeholders to set goals for controlling diarrhea in children; for example, ministries of health and international health agencies could meet and agree on targets for each of the indicators and use the scorecard to track progress over time.

The London Declaration on Neglected Tropical Diseases has recently adopted a similar scorecard to track progress toward 2020 goals to combat neglected tropical diseases [17]. The declaration was launched in January 2012 and the first annual report, published in January 2013, shows how countries are progressing [17]. A press release publicizing the scorecard noted that “the London Declaration Scorecard captures progress made and where efforts must improve if partners hope to reach the 2020 goals. In addition, the scorecard and report set benchmarks for success in 2013 and beyond that would help put the world on a steady trajectory toward those goals” [36]. We believe that a diarrhea control scorecard could play a similar role in putting the world on a “steady trajectory” towards ending avertable childhood diarrhea deaths.

Second, our study found that a scorecard could guide diarrhea control strategy by identifying “positive deviants” as models for success. For example, zinc coverage for children with diarrhea is currently less than 1% in most developing countries, but it is much higher (23%) in Bangladesh (Table 5) [5]. Similarly, ORS coverage in Bangladesh is almost double the coverage levels of the second-best performing country, Tanzania [5]. Thus, Bangladesh, as a “high performer,” would clearly stand out among other countries on a diarrhea scorecard in a way that could stimulate interest in the country’s approach to increasing zinc and ORS coverage. Caution would be needed, however, in assuming that one country’s success in scaling up an intervention would automatically translate well to other settings. For example, in their analysis of the lessons learned from national scale-up of zinc in Bangladesh, the Scaling Up of Zinc for Young Children (SUZY) project team stated that: “The application of a constraints framework is particularly important in the dissemination of scale up process activities because outcomes alone do not measure the transferability of large, complex programs aiming to bring interventions to scale in resource-deprived settings” [37].

Third, it appears that a policy “window of opportunity” has recently opened for launching a diarrhea scorecard. “Policy windows” are agenda-setting opportunities that open at certain moments of time [38]. According to Kingdon’s “three streams” model of policymaking, “Separate streams come together at critical times. A problem is recognized, a solution is developed and available in the policy community, a political change makes it the right time for policy change, and potential constraints are not severe” [38]. Policy windows are opportunities for bringing these streams together in a way that leads to policy action. As described below, a series of events that occurred from 2010–2012, involving new financial commitments and ambitious new goals for child health, have opened up a policy window for launching a diarrhea scorecard.

In September 2010, the Every Woman Every Child Initiative was launched at the UN Millennium Goals Summit, during which US $40 billion was pledged towards improving women’s and children’s health [39]. In February 2012, the Clinton Health Access Initiative and the UCSF Global Health Group reported in the *BMJ* that “for the first time, the 10 countries with the highest burden of diarrhoea have developed ambitious plans to scale-up coverage of effective treatments of diarrhea and pneumonia” [40]. In March 2012, a new UN Commission on Life-Saving Commodities for Women and Children was established, which now advocates for increased access to ORS and zinc, among other essential medicines and commodities [41]. And in June 2012, new pledges were made, and international partnerships forged, at the Child Survival Call to Action summit to “reduce child mortality to below 20 child deaths or fewer per 1,000 live births in every country by 2035” [42], [43]. A scorecard could help to create accountability and transparency around these new scale-up goals and commitments.

We believe that the case for adopting a global diarrhea control scorecard is a strong one. Nevertheless, we acknowledge that our policy suggestion remains an early one and there are many steps that would need to be taken if a scorecard is to become widely used. Our initial prototype will need to be debated and scrutinized by the global health community and, as mentioned, it will need validation in “real world” settings. It will need further refinement through interviews with ministers of health. It will need a “champion” or “champions”—that is, one or more advocates in the child health community who see the value of a diarrhea control scorecard and who work to publicize it and push it to the top of the agenda. Widespread buy-in will be needed from ministers of health of countries with a high burden of diarrheal disease and from international actors, including multilateral agencies and donors. A concerted international effort will be required to boost national capacity for collecting better data on intervention coverage and burden of disease. While these steps present a formidable challenge, the end result of adopting a scorecard could be a dramatic reduction in childhood deaths, an enormously valuable public health pay-off.

## Supporting Information

Text S1 **Semi-structured interview guide.**
(DOCX)

Text S2 **Indicator questionnaire sent to KIs.**
(DOCX)

Table S1 **Basic demographic information about key informants.**
(DOCX)
