# Supplementary material for: Developing a Scorecard to Assess Global Progress in Scaling Up Diarrhea Control Tools: A Qualitative Study of Academic Leaders and Implementers
Source: PLoS One. 2013 Jul 9;8(7):e67320. doi: 10.1371/journal.pone.0067320 (PMC3706531; doi:10.1371/journal.pone.0067320)
Supplement: Text S1 — Semi-structured interview guide. (DOCX) [file pone.0067320.s001.docx]

Semi-Structured Interview Guide:

In your view, what have been the biggest obstacles to combating childhood diarrhea?

What are the most important interventions for reducing child mortality attributed to diarrhea?

What is your view on scorecards in global health (i.e. initiatives which monitor or evaluate countries or agencies on how well they are doing at, say, tackling a disease or improving their accountability)? For example, the Access to Medicine Index gives pharmaceutical companies a composite “score” and ranking according to 106 indicators on efforts to increase access to medicine for societies in need.

Do you think that a diarrhea control scorecard could be valuable?

Which indicators or measures do you believe should be included in a diarrheal disease scorecard?

Who might be interested in a scorecard of diarrhea control?

How might the scorecard be used?

What do you think would be some challenges to using such a scorecard?

What might be some strategies to increase exposure of the scorecard to key stakeholders in global health?

Design of the scorecard (i.e. selection of indicators and metrics, etc.) will be based on a literature review and interviews with experts. Are there any articles or studies that you would recommend for my literature review?

Are there any experts in diarrhea control or public health that you recommend that I interview?
