# Supplementary material for: Developing a Scorecard to Assess Global Progress in Scaling Up Diarrhea Control Tools: A Qualitative Study of Academic Leaders and Implementers
Source: PLoS One. 2013 Jul 9;8(7):e67320. doi: 10.1371/journal.pone.0067320 (PMC3706531; doi:10.1371/journal.pone.0067320)
Supplement: Text S2 — Indicator questionnaire sent to KIs. (DOCX) [file pone.0067320.s002.docx]

Delphi Process Questionnaire:

During my 21 key informant interviews, I asked each expert to suggest indicators for the discussed scorecard. The objective of the scorecard is to assess how different countries are performing in their diarrhea control efforts. A total of 49 different indicators were suggested, as listed below.

My goal now is to narrow down this long list of indicators; the scorecard “prototype” that I plan to create needs to contain a small enough number of indicators to make it simple, easy to use, and non-burdensome (for example, the ALMA malaria scorecard contains 15 indicators).

Please read through the list of indicators below and choose the 10 indicators that you think should be included in the Global Diarrhea Control Scorecard. Please use a “1” to mark your selections in the “Include” column and choose exactly 10 indicators in total.

Also, please select 10 indicators that you *would certainly exclude* from the Global Diarrhea Control Scorecard. Please use a “1” to mark these selections in the “Exclude” column and choose exactly 10 indicators in total.

The 49 indicators have been grouped into 7 “categories” or “sections” for convenience only. You may pick as many or as few from each grouping as you wish; there is no requirement to select one indicator from each section.

**Water and Sanitation**

| Indicator | Include | Exclude |
| --- | --- | --- |
|  |  |  |
| % of population with access to improved sanitation facilities |  |  |
| % of population practicing open defecation |  |  |
| % of population with access to improved drinking-water |  |  |
| An indicator of hand-washing rates |  |  |
| An indicator on latrine usage |  |  |
| Whether or not a country has implemented a mass media campaign to promote hand-washing |  |  |
| % of schools with access to latrines for boys and girls separately |  |  |
| Proportion of urban households connected to sewage |  |  |
| An indicator of urban versus rural latrine usage |  |  |

Please Continue to the Next Page.

**Oral Rehydration Solution (ORS) and Zinc**

| Indicator | Include | Exclude |
| --- | --- | --- |
|  |  |  |
| Zinc coverage in children under five with diarrhea |  |  |
| ORS coverage in children under five with diarrhea |  |  |
| # of districts where zinc and low-osmolarity ORS are available |  |  |
| % of retailers carrying ORS and zinc |  |  |
| # of local pharmaceutical manufacturers producing zinc and ORS in the country |  |  |
| % of mothers between 15 and 45 who know that zinc and ORS are appropriate treatments for diarrhea |  |  |
| An indicator of zinc availability in the public sector |  |  |
| An indicator of zinc availability in the private sector |  |  |
| Proportion of diarrhea cases seen in health facilities that were treated with both zinc and ORS |  |  |
| Whether or not there are ORS/zinc co-packaged products |  |  |
| % of providers correctly administering or prescribing diarrhea treatment |  |  |

**Vaccines**

| Indicator | Include | Exclude |
| --- | --- | --- |
|  |  |  |
| Year of introduction of pneumococcal vaccine |  |  |
| Pneumococcal vaccine coverage |  |  |
| Measles vaccine coverage |  |  |
| Meningococcal vaccine coverage |  |  |
| Haemophilus vaccine coverage |  |  |
| Influenza vaccine coverage |  |  |
| Pertussis vaccine coverage |  |  |
| Rotavirus vaccine coverage |  |  |

**Child and Maternal Health Indicators**

| Indicator | Include | Exclude |
| --- | --- | --- |
|  |  |  |
| % of under-fives with pneumonia taken to an appropriate healthcare provider |  |  |
| % of under-fives with diarrhea taken to an appropriate healthcare provider |  |  |
| Coverage with exclusive breastfeeding |  |  |
| Bed net coverage among children |  |  |
| PMTCT coverage |  |  |
| Coverage with family planning |  |  |
| Vitamin A coverage in children |  |  |
| Height for age Z score at second birthday |  |  |

Please Continue to the Next Page

**Ministry of Health (MoH) Policies**

| Indicator | Include | Exclude |
| --- | --- | --- |
|  |  |  |
| Whether or not a country has over-the-counter (OTC) status for zinc |  |  |
| Whether or not the MoH plans to introduce rotavirus vaccination |  |  |
| Total funding for diarrhea from government and external donors |  |  |
| An indicator of how well the Water, Sanitation, and Hygiene (WASH) and treatment sectors are integrated |  |  |
| Number of ORS packets funded as a percentage of ORS packets needed to have full coverage |  |  |
| % of MOH workers trained in the new diarrhea management protocols under Integrated Management of Childhood Illness (IMCI) |  |  |
| Number of months with stock outs of ORS and zinc in public health facilities |  |  |
| Presence of community case management protocols for diarrhea that include ORS and zinc |  |  |
| Whether or not the public sector purchases ORS and zinc (versus relying on donations) |  |  |

**Social Determinants of Health**

| Indicator | Include | Exclude |
| --- | --- | --- |
|  |  |  |
| % of women who receive secondary education |  |  |
| % of seats held by women in the national government |  |  |

**Burden of Diarrheal Disease**

| Indicator | Include | Exclude |
| --- | --- | --- |
|  |  |  |
| Diarrhea prevalence |  |  |
| Diarrhea-specific mortality rate |  |  |

END
