# Supplementary material for: Developing a Scorecard to Assess Global Progress in Scaling Up Diarrhea Control Tools: A Qualitative Study of Academic Leaders and Implementers
Source: PLoS One. 2013 Jul 9;8(7):e67320. doi: 10.1371/journal.pone.0067320 (PMC3706531; doi:10.1371/journal.pone.0067320)
Supplement: Table S1 — Basic demographic information about key informants. (DOCX) [file pone.0067320.s003.docx]

| **Table S2: Basic demographic information about key informants. ^a^** | | | |
| --- | --- | --- | --- |
| **Key informant (KI)** | **Current position** | **Papers indexed in PubMed: number of papers and key topics of research** |  |
| KI 1 | Senior technical advisor, child survival at a nonprofit, multilateral health organization headquartered in a HIC. | Approx. 50 papers; treatment of diarrhea in children, micronutrients. |  |
| KI 2 | Academic scientist, director of an international child health initiative at a university in a HIC. | Approx. 115 papers; treatment of diarrhea in children, micronutrients, implementation science, provider and caregiver practices. |  |
| KI 3 | Academic scientist at a university in a HIC. | Approx. 20 papers; treatment of diarrhea in children, micronutrients, diarrhea morbidity and mortality, rotavirus vaccination. |  |
| KI 4 | Academic scientist at a university in a HIC; professor of epidemiology. | Approx. 300 papers; treatment of diarrhea in children, micronutrients, diarrhea morbidity and mortality, zinc. |  |
| KI 5 | Academic scientist at a university in a HIC; director of an institute for global health. | Approx. 70 papers; infectious disease epidemiology. |  |
| KI 6 | Professor of global health at a university in a HIC; health consultant to multilateral organizations. | Approx. 10 papers; priorities and agenda-setting in global health. |  |
| KI 7 | Academic scientist at a university in a HIC; professor of epidemiology. | Approx. 60 papers; water and sanitation, disease surveillance. |  |
| KI 8 | Academic scientist at a university in a HIC; professor of epidemiology. | Approx. 20 papers; infectious diseases, water, and sanitation. |  |
| KI 9 | Senior technical advisor, child survival at a nonprofit, multilateral health organization headquartered in a HIC. | Approx. 40 papers; treatment of diarrhea in children, micronutrients, diarrhea morbidity and mortality, zinc and ORS. |  |
| KI 10 | Senior technical advisor, water, sanitation, and hygiene at a health consulting firm headquartered in a HIC | Approx. 5 papers; treatment of diarrhea in children, micronutrients. |  |
| KI 11 | Senior technical advisor, child survival at a nonprofit, multilateral health organization headquartered in a HIC. | Approx. 130 papers; treatment of diarrhea in children, micronutrients, infectious disease epidemiology. |  |
| KI 12 | Academic scientist at a university in a HIC; professor of global health and pediatrics. | Approx. 130 papers; treatment of diarrhea in children, micronutrients, diarrhea morbidity and mortality, zinc and ORS. |  |
| KI 13 | Senior technical advisor, child survival at a nonprofit, multilateral health organization headquartered in a HIC. | Approx. 5 papers; treatment of diarrhea in children, micronutrients, zinc and ORS. |  |
| KI 14 | Academic scientist at a university in a HIC; director of an institute for global health. | Approx. 80 papers; water and sanitation, infectious disease epidemiology. |  |
| KI 15 | Academic scientist at a university in a HIC; director of an institute for global health. | Approx. 100 papers; diarrhea morbidity and mortality, cost-effectiveness of control interventions. |  |
| KI 16 | Academic scientist at a university in a HIC; professor of epidemiology. | Approx. 50 papers; water and sanitation. |  |
| KI 17 | Senior technical advisor, child survival at a health consulting firm headquartered in a HIC. | Approx. 10 papers; treatment of diarrhea in children, micronutrients, zinc and ORS. |  |
| KI 18 | Health policy research fellow at a think tank headquartered in a HIC. | Approx. 15 papers; priorities and agenda-setting in global health. |  |
| KI 19 | Academic scientist at a university in a HIC; professor of epidemiology. | Approx. 80 papers; diarrhea, ORS, enteric infections and child development. |  |
| KI 20 | Academic scientist at a university in a HIC; professor of epidemiology. | Approx. 40 papers; water and sanitation. |  |
| KI 21 | Academic scientist at a university in a LMIC; director of an institute for global health. | Approx. 250 papers; treatment of diarrhea in children, micronutrients, diarrhea morbidity and mortality, zinc and ORS. |  |

^a^ To protect KI’s anonymity, identifying information has been removed.
